# Mobile Internet Technologies, Ecological Momentary Assessment, and Intervention—Poison and Remedy for New Online Problematic Behaviors in ICD-11

**DOI:** 10.3389/fpsyt.2020.00807

**Published:** 2020-08-13

**Authors:** Karol Lewczuk, Monika Gorowska, Yonghui Li, Mateusz Kazimierz Gola

**Affiliations:** ^1^Institute of Psychology, Cardinal Stefan Wyszyński University, Warsaw, Poland; ^2^Key Laboratory of Mental Health, Institute of Psychology, Chinese Academy of Sciences, Beijing, China; ^3^Department of Psychology, University of Chinese Academy of Sciences, Beijing, China; ^4^Swartz Center for Computational Neuroscience, Institute for Neural Computation, University of California, San Diego, San Diego, CA, United States; ^5^Institute of Psychology, Polish Academy of Sciences, Warsaw, Poland

**Keywords:** problematic behavior, mobile technologies, addictive behavior, Internet technologies, ecological momentary assessment, ecological momentary intervention

## Introduction

Technological advancements often present new challenges to mental health and well-being while, at the same time, creating the possibility for new, effective interventions for its preservation, improvement, and recovery (1). In the current commentary, on one hand, we discuss the category of problematic behaviors for which mobile internet technologies have created an outlet. We also consider research challenges related to the conceptualization of these problems, as proposed by the World Health Organization (WHO) for the upcoming 11^th^ revision of International Classification of Disorders (2). On the other hand, we discuss what the development of mobile and online technologies offers for solving or mitigating these problems. Specifically, we focus on ecological momentary assessment (EMA) and intervention (EMI) methodologies (3, 4) and how they can help in overcoming difficulties currently faced in problematic online behavior research, diagnosis, and therapy.

## New Arenas for Problematic and Addictive Behavior

High-speed internet that can be accessed cheaply, at whim, using convenient pocket-sized portable devices and through a multitude of entertaining applications have created a new environment in which gratifying behavior can be easily engaged in and repeated, leading in some cases to the development of detrimental habits. In this way, some online-mediated activities, like cybersex and pornography use, gambling, gaming, buying, social networking, video streaming, or general internet use, can become problematic and—for a subset of users—constitute a mental health problem (5–7). Although some of these behaviors were potentially problematic before the Internet era, the advent of high-speed Internet and widespread use of mobile technology has dramatically increased their addictive potential, making them more significant mental-health threats both on a personal and societal level (8, 9).

## ICD-11 and Problematic Online Behaviors

To address clinical concerns on growing societal significance of new behavioral problems related to the development of technology, WHO recently classified some of them as new diagnostic entities in the ICD-11 (2). Pathological gambling, as well as pathological gaming were described in the “Disorders due to substance use and addictive behaviors” category, while Compulsive Sexual Behavior Disorder (CSBD) was deemed a member of “Impulse Control Disorders” (2, 10), although the discussion on the addictive, compulsive, and/or impulsive roots of the disorder is still ongoing (11–13). An alike debate for some of the other problematic behavior types, e.g., gambling (14, 15), gaming (16) or buying (17, 18) is still in progress.

In our opinion, this discussion raises important questions on determinants for the classification of problematic behavior. Why are some of them classified as addiction-type disorders while others as impulse control disorders? It is worth mentioning that in the Diagnostic and Statistical Manual IV-TR (19) gambling disorder was classified as an impulse control disorder and in the 5^th^ edition (20) as an addiction. A similar change occurred between ICD-10 (21) and ICD-11 (3) for pathological gambling. It raises further important questions: what are the main mechanisms underlying problematic gaming, gambling, or CSBD, and are they homogeneous within each unit? Recognizing these pathological behaviors as psychiatric conditions naturally require further research on their accurate conceptualization and development of effective treatments. Here, another significant question emerges: how do we examine the effectiveness of the treatment? In the case of substance use disorders, there is plenty of objective measures of abstinence such as urinal, saliva, or blood tests (22, 23). In contrast, there is no such objective and retrospective method of assessment for problematic behaviors. However, if the behavior is engaged in online, reliable tracking of activity can be made possible by using EMA.

## Ecological Momentary Assessment: A Way to Better Understand New Problematic Behaviors

EMA is a method delivered through mobile devices aimed to collect and record a person’s activities and inner states in real time as they occur, by periodically prompting the user to fill-out short assessment questionnaires (3, 24, 25). Among the most appreciated benefits of EMA are: (a) minimization of recall bias by assessing the current, instead of retrospective data; (b) maximization of ecological validity through data collection in a real-world setting, as opposed to data collection in controlled laboratory environments; (c) enabling to gather a large amount of quantitative data from individuals across time and different contexts (3); as well as (d) to identify the dynamic interplay between the variables, thereby helping (e) to infer causal relationships between them (26). Most recent versions of EMA running on new mobile devices equipped with sophisticated sensors and features allow for real-time geolocation, active tracking, objective inferring on stress or arousal level based on biosensors (e.g., heart rate and temperature measurements with smartwatches) and tracking of actual online behavior (e.g., on smartphone or tablet) (3, 24, 25). Recent advances in the research have transformed EMA from an initially a valuable data collection method into real-time intervention tool - EMI (5), providing not only the assessment but also the management of momentary variables (22, 27, 28). In our opinion research involving EMA may help (a) to solve the etiological debate on the correct conceptualization and behavioral phenotyping of gambling, gaming, CSBD, and not-yet classified behaviors such as problematic social media use of video binging. (b) It provides a more reliable and objective measure of frequency and severity, progression, or improvement of actual behaviors; and in combination with EMI (c) can offer new scientifically verified treatments.

## Addictive, Impulsive, and Compulsive Models: Solving the Puzzle

To illustrate the benefits of using EMA in advancing the debate between addictive, impulsive, and compulsive models of online problematic behaviour, we will use the example of CSBD, for which the discussion is especially lively (11). The validity of each model is based on the presence and relative importance of symptoms predicted by each of the three models (29). The presence of obsessions driving sexual behavior, as well as its relative rigidity and ritualism, can indicate its kinship to obsessive-compulsive disorders and support the validity of the compulsive model. The presence of withdrawal symptoms and tolerance lends support to the addictive model, while impulsive sexual behavior-driven mainly by pleasure-seeking with associated general impulse control deficits indicates the validity of the impulsive model (12, 13, 29, 30). Compared to a standard self-report method, EMA can be better suited to investigate these predictions and the concurrent validity of these models because: (a) it enables ecologically valid measurements of symptom feature predicted by the three models, which is especially important as addictive behavior have a highly contextual character; (b) phenomena-like obsessions, withdrawal symptoms and cravings are transient states and the adopted method of measurement should be able to reflect their fluctuation (which is hard to achieve with retrospective, aggregate measurements); (c) frequency of a targeted problematic behavior can be assessed with higher accuracy, using ecological declarative measurements or objective indicators (see the section below); (d) multiple points of measurement over time allow for directional relationships between variables to be investigated; (e) Lastly, the most valid solution to the debate on etiology may not rely on singling out the model that is the best universal descriptor of symptoms for all subjects, but on investigating possible subtypes or profiles of the disorder that pertain to the three described models and the prevalence of these subtypes (allowed due to the possibility for gathering large amounts of data with EMA) (31). In this way, EMA can provide unique contributions to an accurate classification of gambling, gaming, CSBD, as well as problematic behavior not yet classified (e.g., problematic buying, social networking), which are harder to obtain using more traditional approaches.

## For Online Problematic Behavior, Accurate Behavior Tracking Is Crucial

Self-reports are associated with numerous errors, related to faulty memory, memory bias, or social desirability bias (32). We have evidence of inaccuracies of self-reports regarding problematic behaviors like gambling (33) or gaming (34). Moreover, these biases can be the strongest for the most active/problematic users (33). This is especially important, as for online problematic behavior the frequency of the behavior is one of the main factors contributing to the severity of experienced negative symptoms (35). With respect to this, EMA delivered on smartphones offers unique advantages, as the methodology allows for more accurate tracking of frequencies of a target behavior, thoughts or moods, with higher compliance and lower data loss compared to standard paper-pencil or computer-based questioners or diaries (36). Additionally, in conjunction with biosensors and specific software solutions, it enables objective data collection continuously and passively, with little or no burden to participants (heart rate, temperature, location, smartphone use, or social media and online engagement gathered through data mining), which provide reliable diagnostic and predictive biomarkers of examined constructs (25, 28, 37). Future studies in online problematic behaviors may combine both subjective and objective measurements that help to accurately assess behavioral and psychological changes over time and between contexts as well as to better monitor progression, recovery, and possible relapses in problematic behavior treatment (28, 37, 38). Although objective tracking of problematic behavior in its offline form is harder, EMA can still provide more accurate assessments based on subjective indicators (36). Additionally, as (a) offline vs online forms of problematic activities can have differing features, patterns of use, as well as risk and protective factors; (b) subjects engaging in online vs offline problematic behaviors can have different characteristics; (c) similar differences can potentially appear depending on the used device [e.g., computer vs mobile mediated form of problematic behavior, see examples for problematic gambling (39–42)], EMA/EMI can be employed as a useful tool for investigating these differences.

## EMI as a Promising Method for Addictive Behavior Intervention

A significant gap between the number of people that need or could benefit from treatment and the number of people actually receiving it is one of the most urgent problems in therapy (43). Due to its high cost-effectiveness, EMI offers the possibility to improve access to evidence-based treatment for various populations, democratizing it (1, 5). EMI seems to be promising owning the possibility of identifying contextual (social interaction, location) and intra-individual (craving, mood, physiological responses) precipitating factors of lapses through the employment of machine learning algorithms and data mining (44, 45). EMI has the potential to tailor the intervention to the demographic, psychological, and behavioral characteristics of a person and specific symptoms experienced (46), meta-analytic evidence shows that such adaptive features increase the effectiveness of the interventions (47). As online problematic behavior can be induced by external cues, delivering just-in-time adaptive interventions (46) can contribute to successful behavioral management in many cases. Recent research has shown that physiological information gathered with EMA can be positively used for EMI (e.g., when the stress indicators exceed a threshold value, relaxation exercises can be prompted) (48). Moreover, EMI allows for the preservation of anonymity, therefore helping to overcome the fear of social-stigmatization (49). Lastly, EMI is well suited for the group of people manifesting online problematic behavior as a barrier to entry does not exist: members of this group are used to smartphones and mobile applications.

Additionally, it is worth underlying that the discussed methodology has already proven to be useful in assessment and therapy of substance addictions and related behavior: aiding recovery from alcoholism by reducing risky drinking episodes (50), helping to limit binge drinking among young people (51), supporting smoking cessation (52), reducing marijuana cravings (53), and significantly predicting substance use relapse after treatment (54).

Despite these factors, to the best of our knowledge, and recent-meta-analytic work (55), no randomized controlled trials for interventions delivered *via* smartphones are available for online problematic behavior. Supplementing this lack in the near future is key for further advancements.

## New Ethical Issues

Although tracking objective indicators of online behavior and offering tailored interventions are possible with mobile devices, it raises ethical issues connected to gathering, managing, and storing sensitive, personal data (56). Also, using mobile applications as the method of delivery of interventions can potentially result in increasing, not decreasing, the reliance of a person on technology (2). Researchers and commentators now point to the fact that in order to attract users and extend time online, application creators often make them, in fact, more addictive (57), which results in a dangerous possibility of exchanging one addiction to another in the process of treatment (3). Repetitive assessment of problematic behavior and associated variables can make the behavior itself more salient and increase the risk of relapse or increase craving, which has to be monitored and taken into account by researchers and developers (58). Overall, there is an existing need to improve the evidence base behind mobile app products through careful evaluation of their safety and effectiveness before the public distribution or clinical use (59).

## Conclusion

The development of mobile and online technologies has allowed for the proliferation of online problematic behavior, but can also be harnessed for more effective intervention and therapy, as well as effective tackling of remaining theoretical questions. Although challenges exist (e.g., ethical issues), EMA and EMI methodologies seem to have huge potential for online problematic behavior research and therapy, which is—as yet—untapped.

## Author Contributions

Review of the literature was conducted by KL and MG. The manuscript draft was developed by KL, MG, and MKG. All authors contributed to the article and approved the submitted version.

## Funding

YL is supported by National Key Research and Development Program of China (2017YFC1310405) and the National Natural Science Foundation of China (U1736124). MKG is supported by the Bekker programme grant of the Polish National Agency for Academic Exchange (PPN/BEK/2019/1/00245/U/00001).

## Conflict of Interest

The authors declare that the research was conducted in the absence of any commercial or financial relationships that could be construed as a potential conflict of interest.
